# Reversing autism by targeting downstream mTOR signaling

**DOI:** 10.3389/fncel.2013.00028

**Published:** 2013-03-26

**Authors:** Hansen Wang, Laurie C. Doering

**Affiliations:** ^1^Faculty of Medicine, University of TorontoToronto, ON, Canada; ^2^Department of Pathology and Molecular Medicine, Faculty of Health Sciences, McMaster UniversityHamilton, ON, Canada

**Figure 1:**
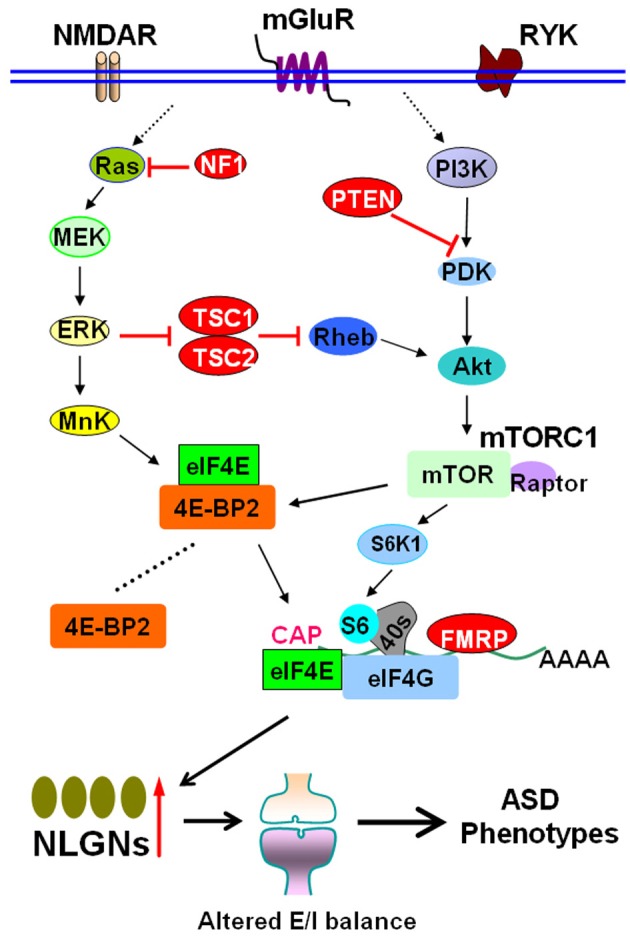
**The mTOR signal pathway in autism spectrum disorders**. The mTOR pathway integrates inputs from different sources, such as NMDAR, mGluR, and RYK. Activation of mTORC1 promotes the formation of the eIF4F initiation complex. Mutations in *TSC1*/*2*, *NF1*, and *PTEN*, or loss of FMRP due to mutations of the *FMR1*gene, cause hyperactivity of mTORC1–eIF4E pathway and lead to syndromic ASDs. 4E-BP2 inhibits translation by competing with eIF4G for eIF4E binding. Gkogkas et al. demonstrated that removal of 4E-BP2 or overexpression of eIF4E enhances cap-dependent translation. The increased translation of NLGNs causes increased synaptic E/I ratio, which may eventually lead to ASD phenotypes. Abbreviations: Akt, also known as PKB, protein kinase B; ASD, autism spectrum disorder; 4E-BP2, eIF4E-binding protein 2; E/I, excitation/inhibiton; ERK, extracellular signal regulated kinase; FMRP, fragile X mental retardation protein; MEK, mitogen-activated protein/ERK kinase; mGluR, metabotropic glutamate receptor; mTOR, mammalian target of rapamycin; mTORC1, mTOR complex 1; *NF1*, neurofibromatosis 1; NLGN, neuroligin; NMDAR, NMDA receptor; PDK, phosphoinositide dependent kinase; PI3K, phosphoinositide-3 kinase; *PTEN*, Phosphatase and tensin homolog; Raptor, regulatory associated protein of mTOR; Rheb, Ras homolog enriched in brain; RYK, receptor-like tyrosine kinase; S6K1, p70 ribosomal S6 kinase 1; *TSC*, tuberous sclerosis complex.

Autism spectrum disorders (ASDs) are a group of clinically and genetically heterogeneous neurodevelopmental disorders characterized by impaired social interactions, repetitive behaviors and restricted interests (Baird et al., 2006; Zoghbi and Bear, 2012). The genetic defects in ASDs may interfere with synaptic protein synthesis. Synaptic dysfunction caused by aberrant protein synthesis is a key pathogenic mechanism for ASDs (Kelleher and Bear, 2008; Richter and Klann, 2009; Ebert and Greenberg, 2013). Understanding the details about aberrant synaptic protein synthesis is important to formulate potential treatment for ASDs. The mammalian target of the rapamycin (mTOR) pathway plays central roles in synaptic protein synthesis (Hay and Sonenberg, 2004; Hoeffer and Klann, 2010; Hershey et al., 2012). Recently, Gkogkas and colleagues published exciting data on the role of downstream mTOR pathway in autism (Gkogkas et al., 2013) (Figure 1).

Previous studies have indicated that upstream mTOR signaling is linked to ASDs. Mutations in tuberous sclerosis complex (*TSC*) *1*/*TSC2*, neurofibromatosis 1 (*NF1*), and Phosphatase and tensin homolog (*PTEN*) lead to syndromic ASD with tuberous sclerosis, neurofibromatosis, or macrocephaly, respectively (Kelleher and Bear, 2008; Bourgeron, 2009; Hoeffer and Klann, 2010; Sawicka and Zukin, 2012). TSC1/TSC2, NF1, and PTEN act as negative regulators of mTOR complex 1 (mTORC1), which is activated by phosphoinositide-3 kinase (PI3K) pathway (Kelleher and Bear, 2008; Auerbach et al., 2011; Sawicka and Zukin, 2012) (Figure 1). Activation of cap-dependent translation is a principal downstream mechanism of mTORC1. The eIF4E recognizes the 5′ mRNA cap, recruits eIF4G and the small ribosomal subunit (Richter and Sonenberg, 2005; Hershey et al., 2012). The eIF4E-binding proteins (4E-BPs) bind to eIF4E and inhibit translation initiation. Phosphorylation of 4E-BPs by mTORC1 promotes eIF4E release and initiates cap-dependent translation (Richter and Klann, 2009; Hoeffer and Klann, 2010) (Figure 1). A hyperactivated mTORC1–eIF4E pathway is linked to impaired synaptic plasticity in fragile X syndrome, an autistic disorder caused by lack of fragile X mental retardation protein (FMRP) due to mutation of the FMR1 gene (Wang et al., 2010; Auerbach et al., 2011; Santoro et al., 2012; Wang et al., 2012), suggesting that downstream mTOR signaling might be causally linked to ASDs. Notably, one pioneering study has identified a mutation in the EIF4E promoter in autism families (Neves-Pereira et al., 2009), implying that deregulation of downstream mTOR signaling (eIF4E) could be a novel mechanism for ASDs.

As an eIF4E repressor downstream of mTOR, 4E-BP2 has important roles in synaptic plasticity, learning and memory (Banko et al., 2005; Richter and Klann, 2009). Writing in their Nature article, Gkogkas and colleagues reported that deletion of the gene encoding 4E-BP2 (Eif4ebp2) leads to autistic-like behaviors in mice. Pharmacological inhibition of eIF4E rectifies social behavior deficits in Eif4ebp2 knockout mice (Gkogkas et al., 2013). Their study in mouse models has provided direct evidence for the causal link between dysregulated eIF4E and the development of ASDs.

Are these ASD-like phenotypes of the Eif4ebp2 knockout mice caused by altered translation of a subset mRNAs due to the release of eIF4E? To test this, Gkogkas et al. measured translation initiation rates and protein levels of candidate genes known to be associated with ASDs in hippocampi from Eif4ebp2 knockout and eIF4E-overexpressing mice. They found that the translation of neuroligin (NLGN) mRNAs is enhanced in both lines of transgenic mice. Removal of 4E-BP2 or overexpression of eIF4E increases protein amounts of NLGNs in the hippocampus, whereas mRNA levels are not affected, thus excluding transcriptional effects (Gkogkas et al., 2013). In contrast, the authors did not observe any changes in the translation of mRNAs coding for other synaptic scaffolding proteins. Interestingly, treatment of Eif4ebp2 knockout mice with selective eIF4E inhibitor reduces NLGN protein levels to wild-type levels (Gkogkas et al., 2013). These data thus indicate that relief of translational suppression by loss of 4E-BP2 or by the overexpression of eIF4E selectively enhances the NLGN synthesis. However, it cannot be ruled out that other proteins (synaptic or non-synaptic) may be affected and contribute to animal autistic phenotypes.

Aberrant information processing due to altered ratio of synaptic excitation to inhibition (E/I) may contribute to ASDs (Rubenstein and Merzenich, 2003; Bourgeron, 2007; Uhlhaas and Singer, 2012). The increased or decreased E/I ratio has been observed in ASD animal models (Chao et al., 2010; Bateup et al., 2011; Luikart et al., 2011; Schmeisser et al., 2012). In relation to these E/I shifts, Gkogkas et al then examined the synaptic transmission in hippocampal slices of Eif4ebp2 knockout mice. They found that 4E-BP2 de-repression results in an increased E/I ratio, which can be explained by the increase of vesicular glutamate transporter and spine density in hippocampal pyramidal neurons. As expected, application of eIF4E inhibitor restores the E/I balance (Gkogkas et al., 2013).

Finally, in view of the facts that genetic manipulation of NLGNs results in ASD-like phenotypes with altered E/I balance in mouse models (Chubykin et al., 2007; Tabuchi et al., 2007; Etherton et al., 2011) and NLGN mRNA translation is enhanced concomitant with increased E/I ratio in Eif4ebp2 knockout mice, Gkogkas et al. tested the effect of NLGN knockdown on synaptic plasticity and behaviour in these mice (Gkogkas et al., 2013). NLGN1 is predominantly postsynaptic at excitatory synapses and promotes excitatory synaptic transmission (Varoqueaux et al., 2006; Kwon et al., 2012). The authors found that NLGN1 knockdown reverses changes at excitatory synapses and partially rescues the social interaction deficits in Eif4ebp2 knockout mice (Gkogkas et al., 2013). These findings thus established a strong link between eIF4E-dependent translational control of NLGNs, E/I balance and the development of ASD-like animal behaviors (Figure 1).

In summary, Gkogkas et al. have provided a model for mTORC1/eIF4E-dependent autism-like phenotypes due to dysregulated translational control (Gkogkas et al., 2013). This novel regulatory mechanism will prompt investigation of downstream mTOR signaling in ASDs, as well as expand our knowledge of how mTOR functions in human learning and cognition. It may narrow down therapeutic targets for autism since targeting downstream mTOR signaling reverses autism. Pharmacological manipulation of downstream effectors of mTOR (eIF4E, 4E-BP2, and NLGNs) may eventually provide therapeutic benefits for patients with ASDs.
